# Supplementary material for: Genetic architecture of spatial electrical biomarkers for cardiac arrhythmia and relationship with cardiovascular disease
Source: Nat Commun. 2023 Mar 14;14:1411. doi: 10.1038/s41467-023-36997-w (PMC10015012; doi:10.1038/s41467-023-36997-w)
Supplement: Supplementary file 1 — Supplementary Information [file 41467_2023_36997_MOESM1_ESM.pdf]

Supplementary Figures

a. spQRSTa

Multi-Ancestry

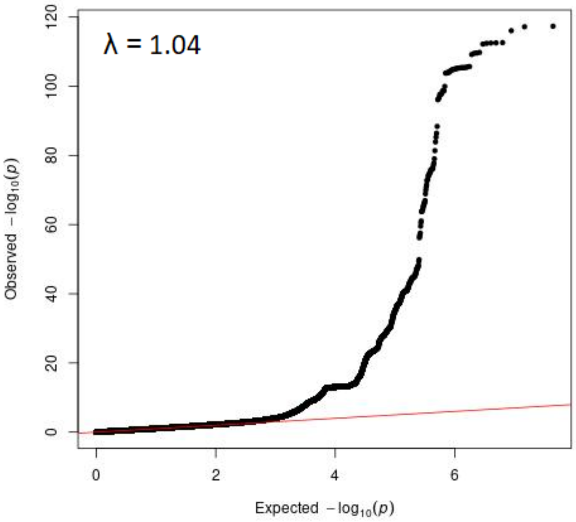

European

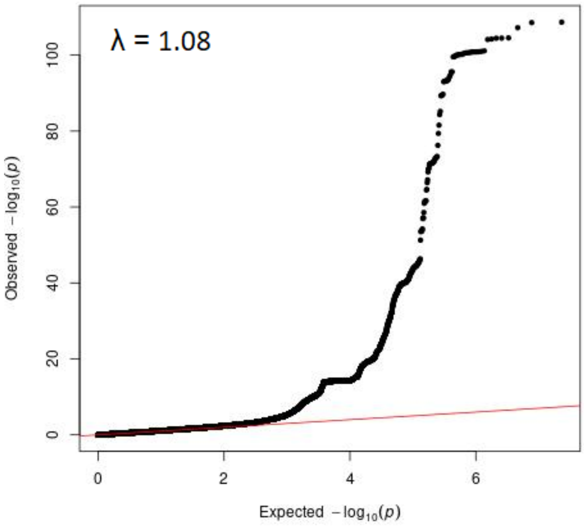

Hispanic/Latino

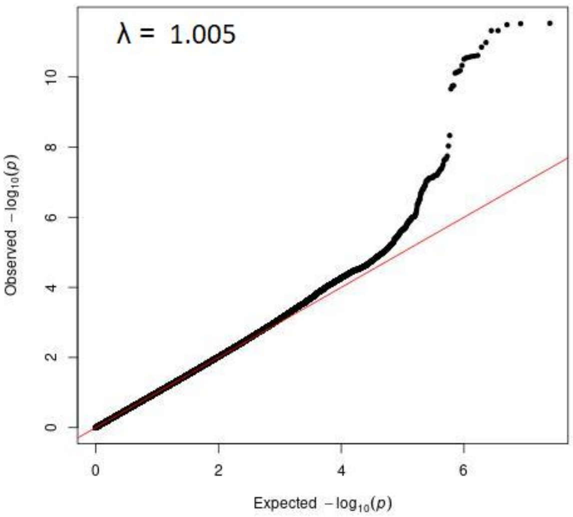

African

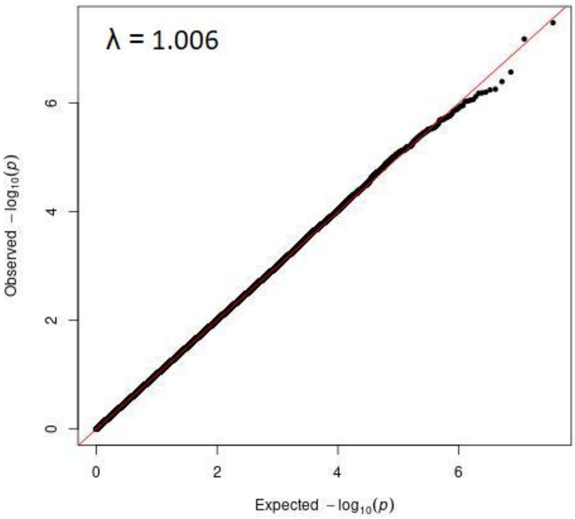

b. fQRSTa

Multi-Ancestry

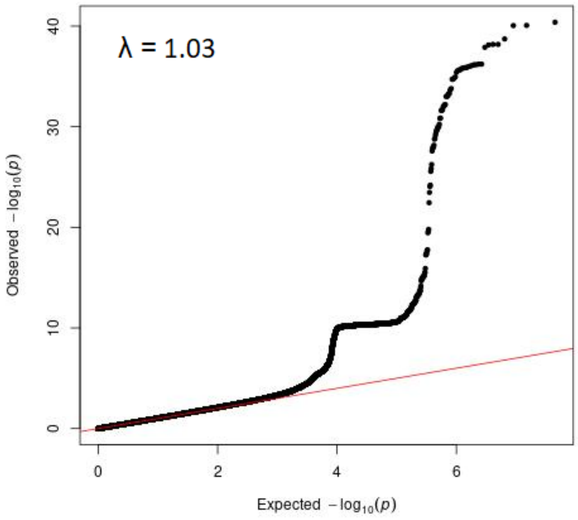

European

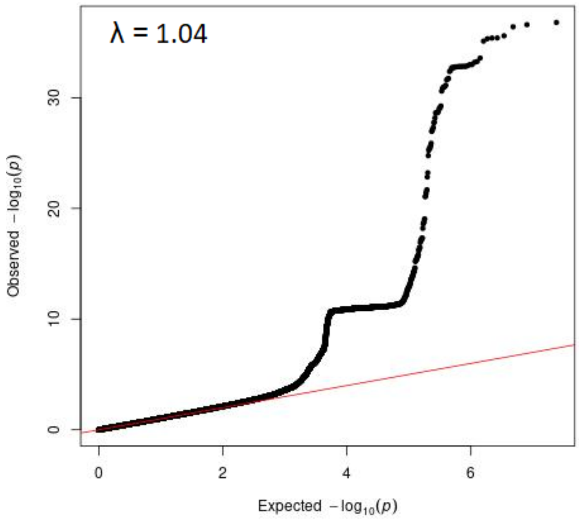

Hispanic/Latino

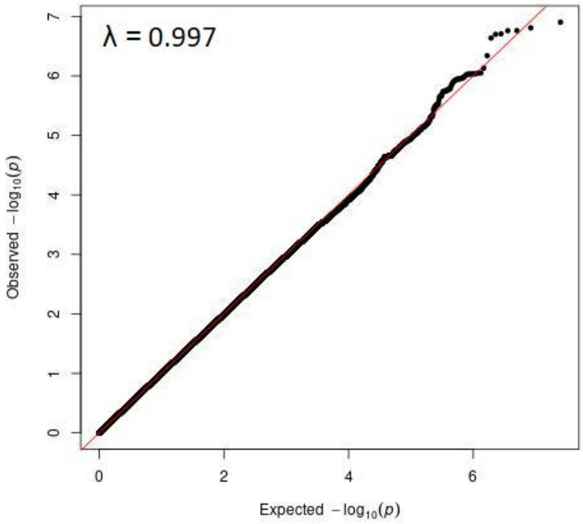

African

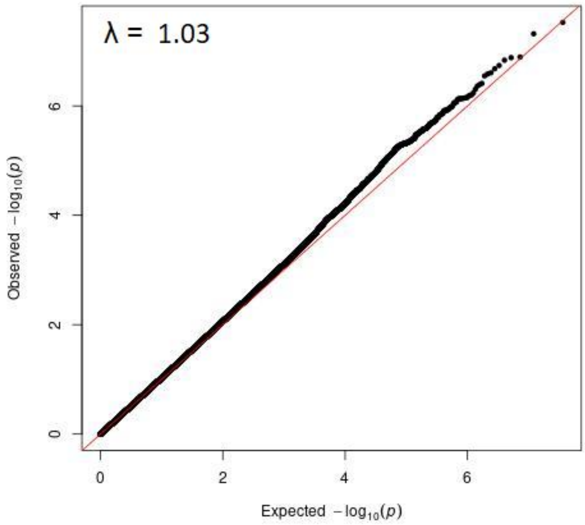

Supplementary Figure 1: Quantile-Quantile plots for spQRSTa and fQRSTa meta-analyses
Quantile-Quantile (QQ) plots for spatial QRS-T angle (spQRSTa) and frontal QRS-T angle (fQRSTa) meta-analyses comparing observed *P*-values (Y-axis) against the expected from a theoretical chi-squared distribution (X-axis). λ = genomic inflation statistic (lambda) (a. spQRSTa, b. fQRSTa). Early deviation from the reference line was evident in multi-ancestry and European ancestry multi-ancestry plots. This was driven by a locus on chromosome 17 (KANSL) that contained a cluster of variants in strong LD with similar *P*-values.

a. spQRSTa

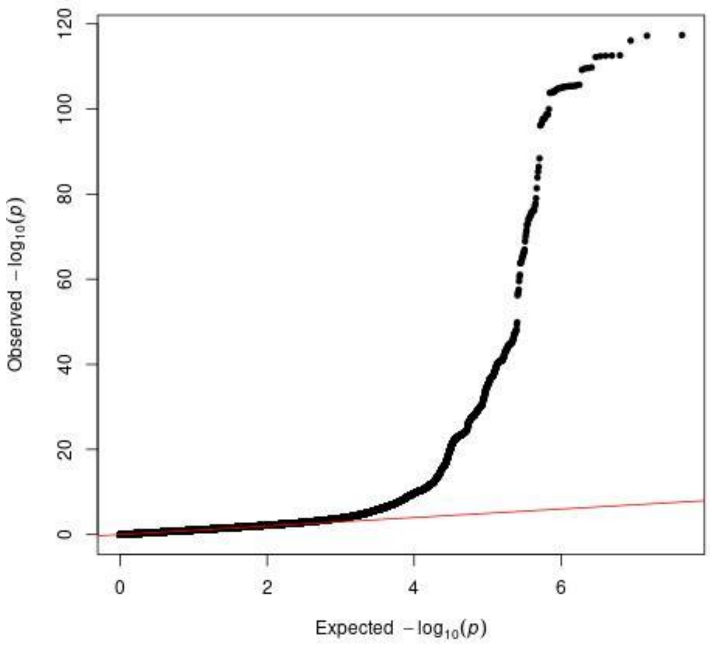

b. fQRSTa

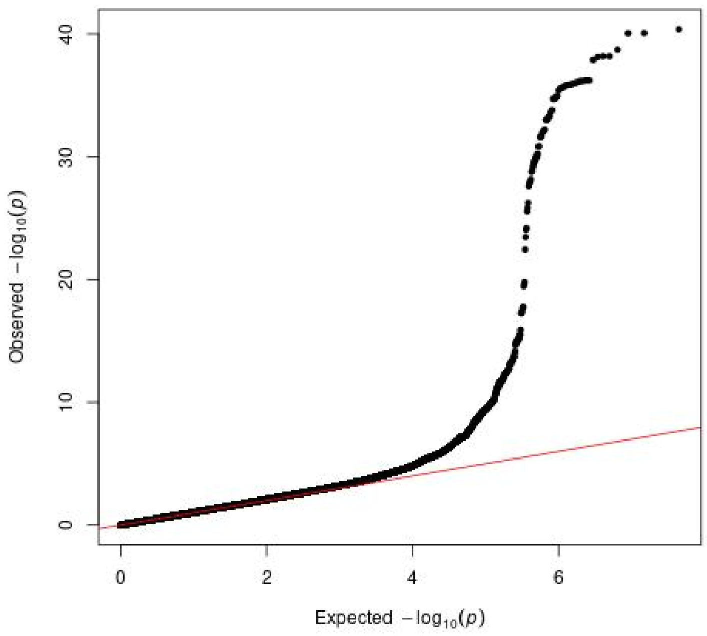

Supplementary Figure 2: Quantile-Quantile plots for spQRSTa and fQRSTa multi-ancestry meta-analyses after exclusion of the chromosome 17 locus *KANSL*

For both spatial and frontal QRS-T angle (spQRSTa and fQRSTa, respectively) multi-ancestry meta-analyses, variants at the chromosome 17 locus (hg19 positions 43006952 to 45221966) were excluded and the Quantile-Quantile plots reproduced (a. spQRSTa, b. fQRSTa). These compare observed *P*-values (Y-axis) against the expected from a theoretical chi-squared distribution (X-axis). The early deviation previously evident (as described in Supplemtary Figure 1) is now no longer present.

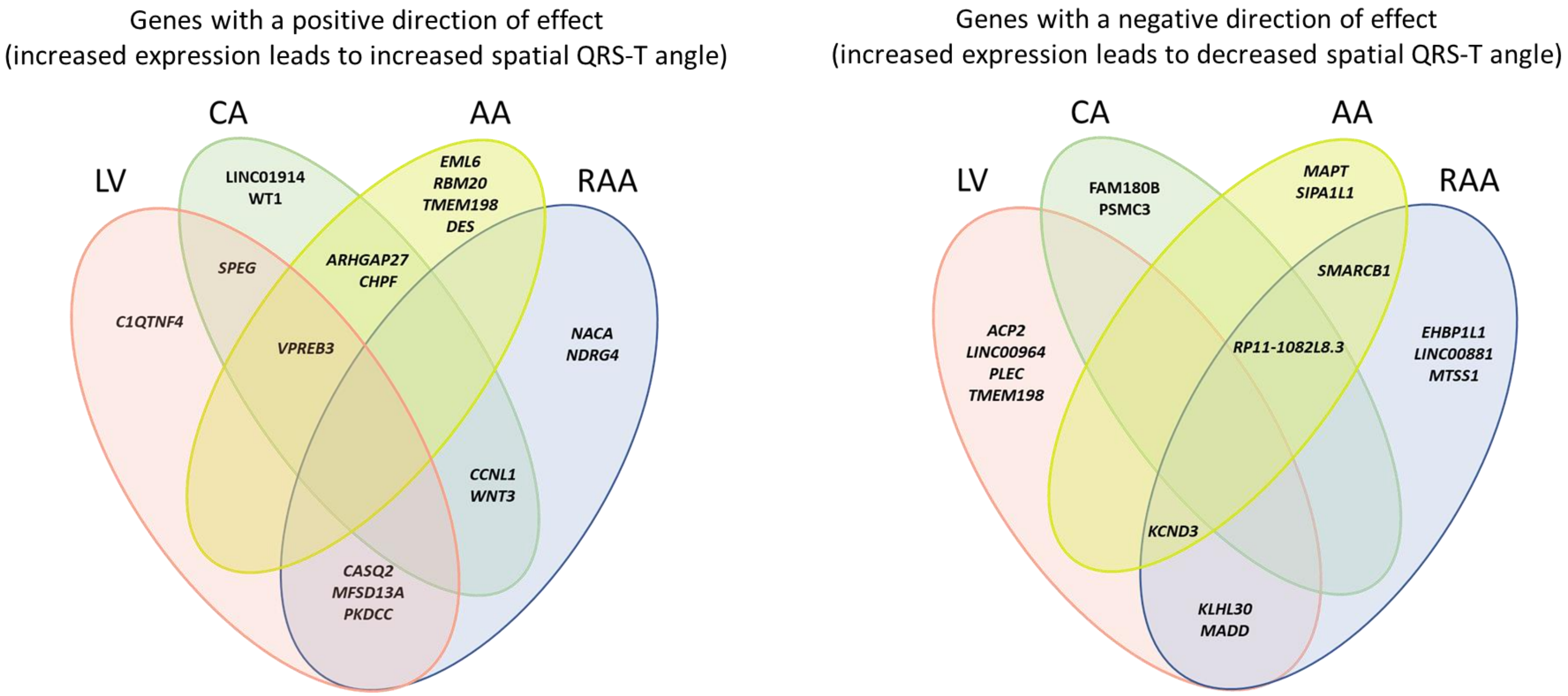

Supplementary Figure 3: Transcriptome-wide association study results for spQRSTa
Venn diagrams showing the overlap of genes in relevant tissues and association of expression with the spatial QRS-T angle (spQRSTa). Left side: Genes where increased expression is associated with an increase in the spQRSTa. Right side: Genes where increased expression is associated with a decrease in the spQRSTa. LV: Left ventricle, RAA: Right atrial appendage, CA: Coronary artery, AA: Aortic artery. Analyses were performed with S-PREDIXCAN software using European ancestry meta-analysis summary statistics.

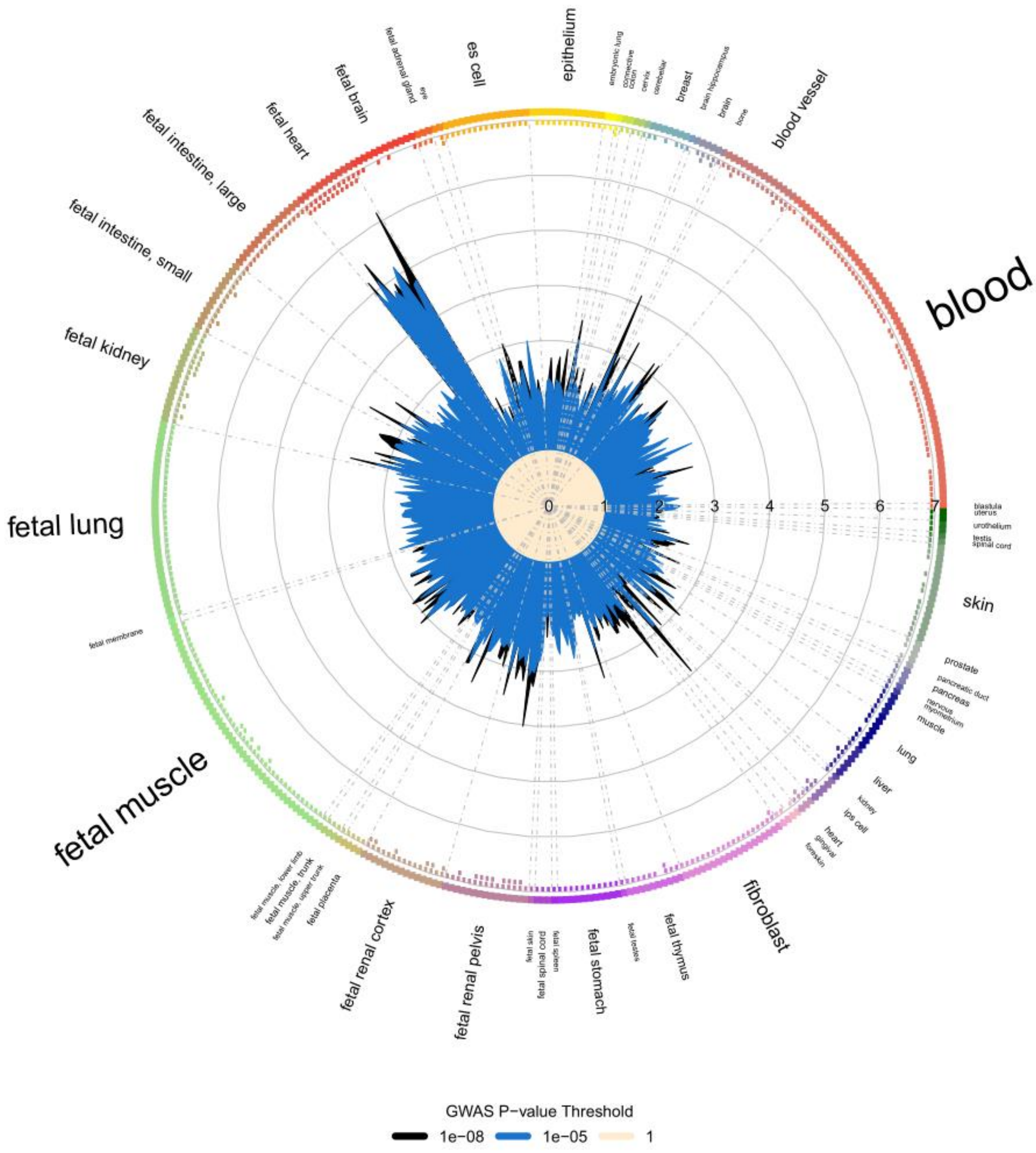

Supplementary Figure 4: Enrichment of spQRSTa variants in DNaseI Hypersensitivity sites

DNaseI hypersensitivity sites were derived from the Encyclopaedia of DNA elements (ENCODE) and Roadmap Epigenomics projects using GARFIELD. European ancestry meta-analysis summary statistics for the spatial QRS-T angle (spQRSTa) were used. Tissue font size is proportional to the number of cell types for that tissue. Radial plot shows odds ratio (OR) in each cell type at two GWAS *P*-value thresholds ($<1x10^{-5}$ and $<5x10^{-8}$) (two-sided statistical tests). Small dots on the inner side of the outer circle show if the observed enrichment is significant in direction outside to inside.

a. spQRSTa

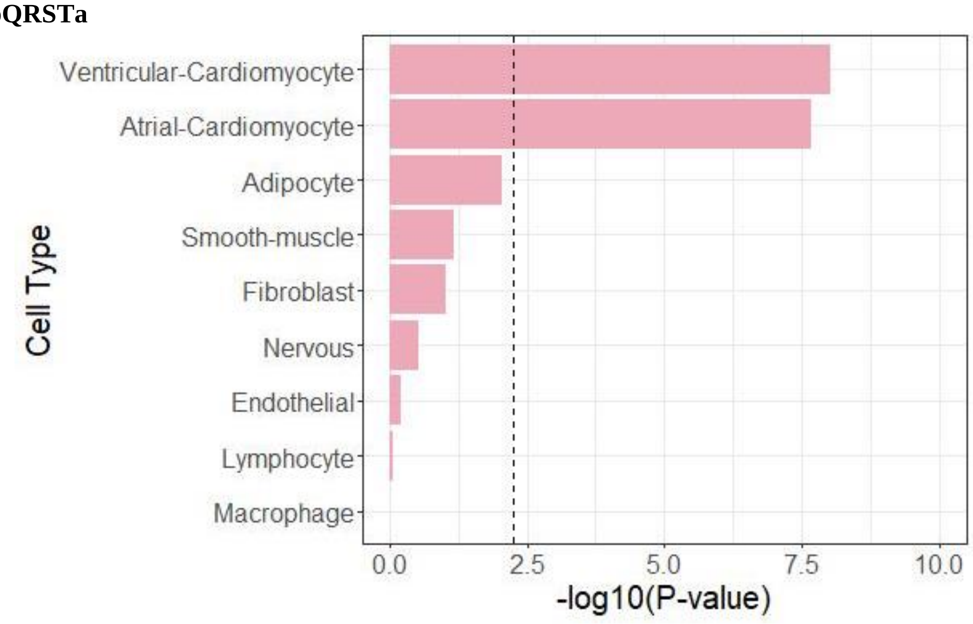

b. fQRSTa

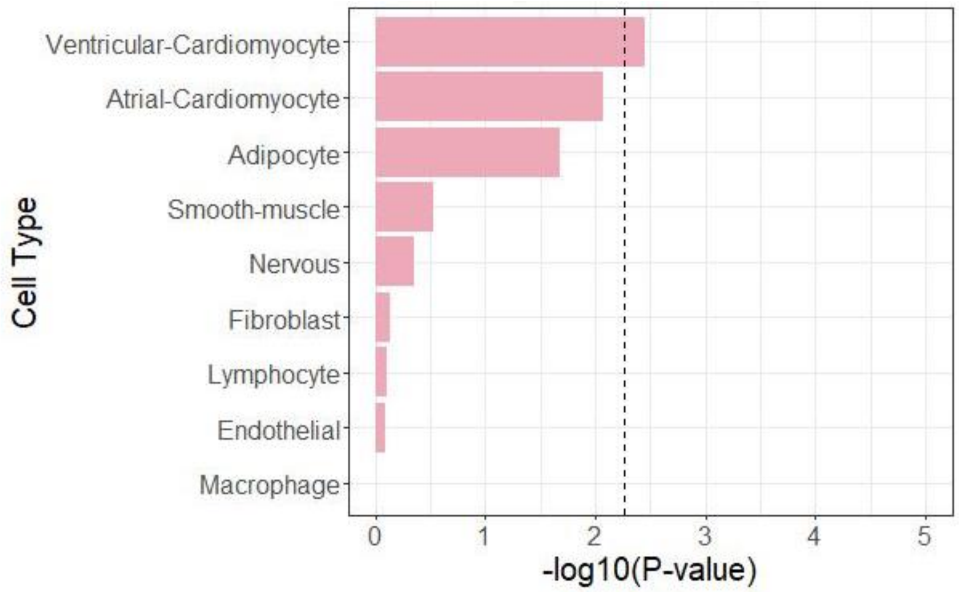

Supplementary Figure 5: Cardiac cell-type specific overlap with snATAC-seq peaks for multi-ancestry meta-analyses

Results for single nuclear ATAC seq (snATAC-seq) analyses for the spatial and frontal QRS-T angle (spQRSTa [a] and fQRSTa [b], respectively) multi-ancestry meta-analyses. X-axis: log base 10 *P*-value for cell-type enrichment. Y-axis: Cell-type. Vertical dashed line: Bonferroni corrected significance threshold for number of cell types used ($0.05/7 = 7.1 \times 10^{-3}$) to declare signifiance. *P*-values were calculated under the null hypothesis (one-sided) that all peak ranks are random.

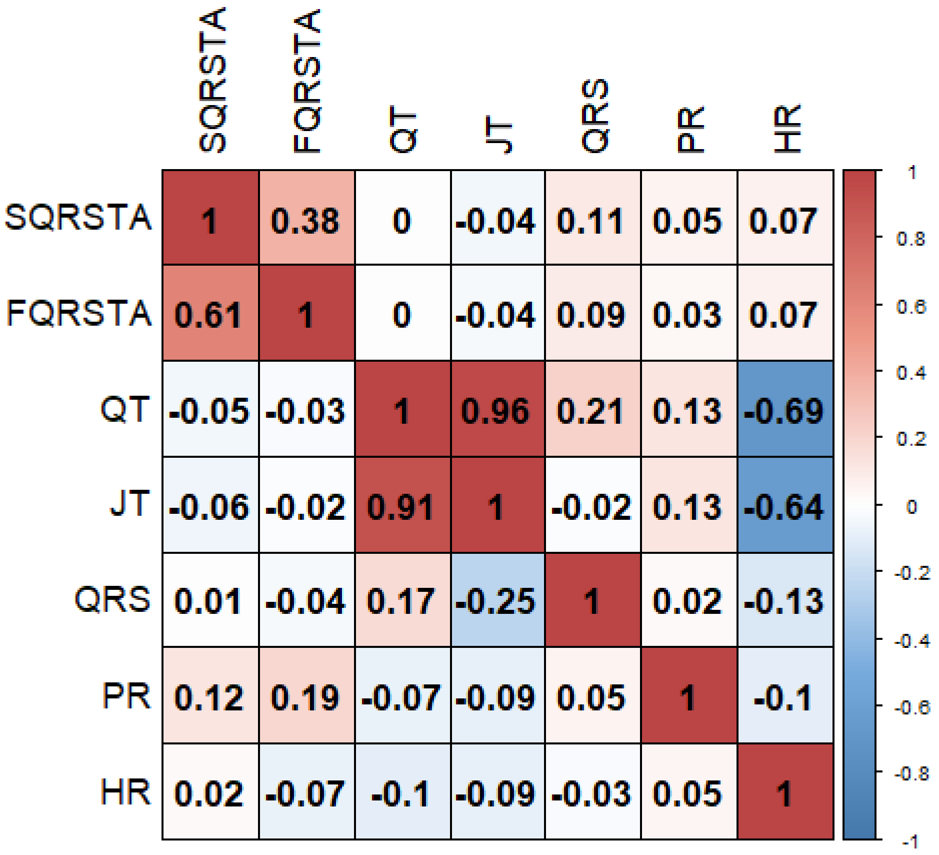

Supplementary Figure 6: Phenotypic and genetic correlation of the spQRSTa and fQRSTa with other ECG measures
Phenotypic correlations calculated in UK Biobank individuals of European ancestry in the top right triangle. All measures were calculated using resting 12 lead ECGs in ~34K individuals of European-ancestry. Spearman's rank (r_s) correlation coefficient is reported. Genetic correlations (r_g) are shown in the lower left triangle using summary statistics from previously reported GWAS meta-analyses. Colors are graded according to the corresponding correlation coefficient with blue for negative correlations and red for positive correlations. spQRSTA; Spatial QRS-T angle, fQRSTA; Frontal QRS-T angle, HR; Heart rate.

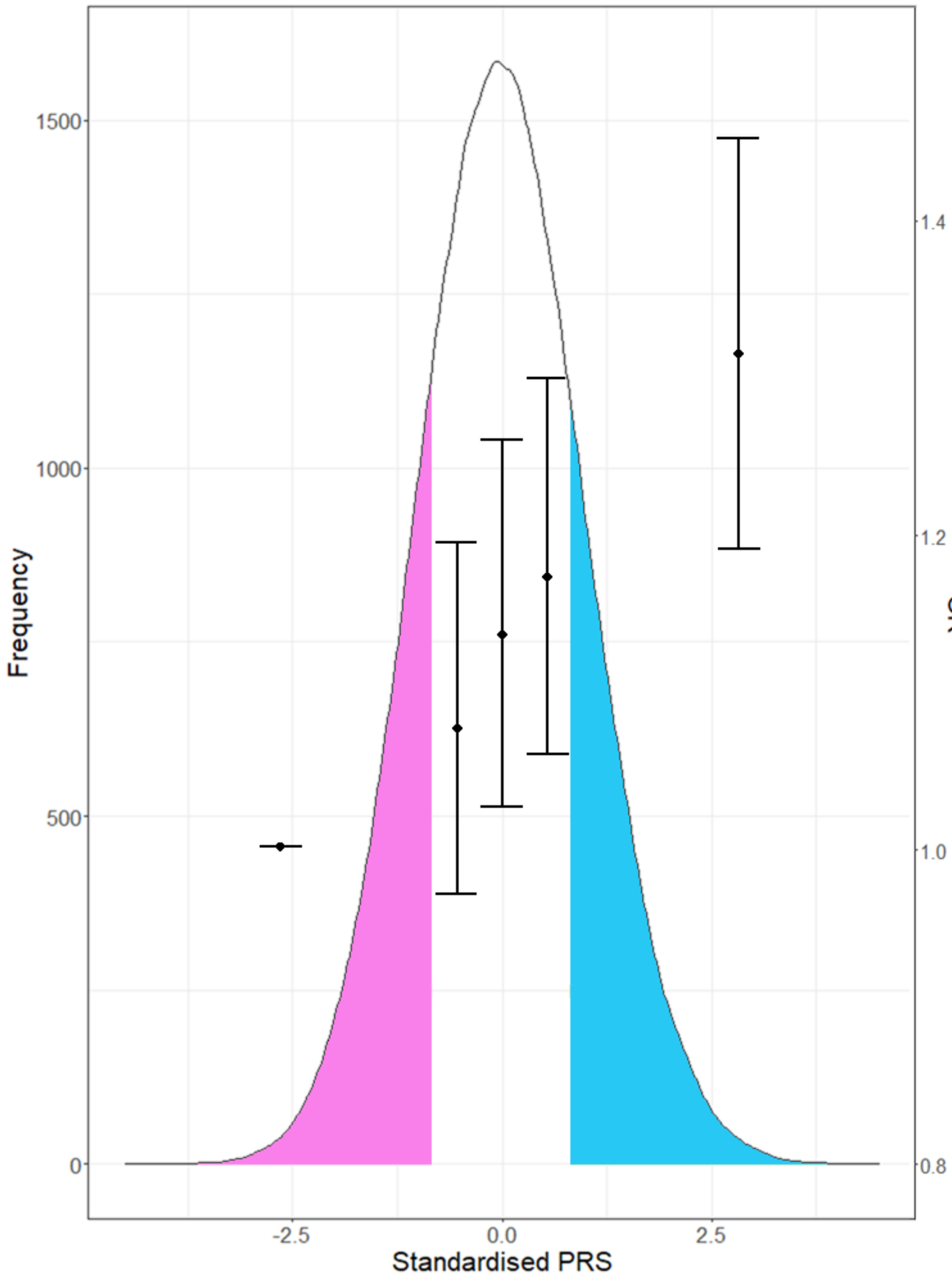

Supplementary Figure 7: Association of genetically determined spQRSTa with fascicular and bundle branch block

Association of genetically determined spatial QRS-T angle (spQRSTa) with prevalent and incident cases of conduction disease in 395,758 unrelated individuals of European ancestry from UK Biobank. X-axis: Polygenic risk score (PRS) standardised to the mean. Y axis left: Number of individuals. Y axis right: OR (Odds ratio), per SD increase in PRS. Graph area: Error bards displace odds ratio and 95% CI for each quintile (Q1-Q5) from left to right.The shaded pink area corresponds to the lowest qunitile (Q1) and the shaded blue area corresponds to the upper quintile (Q5).

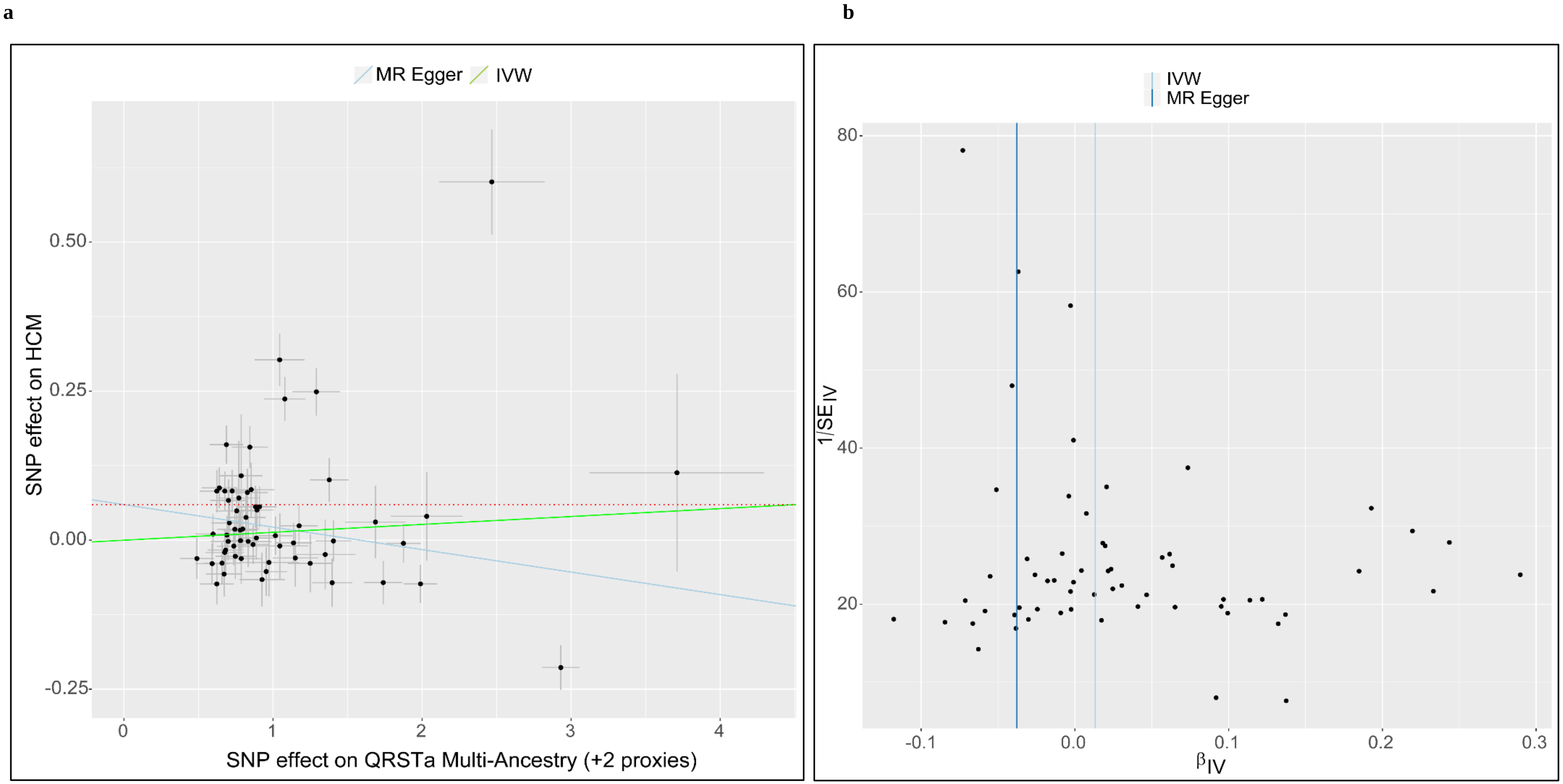

Supplementary Figure 8: Scatter and funnel plots for spQRSTa-HCM multi-ancestry MR analyses.
a. Scatter plot of individual variant regression coefficients with Inverse-variance weighted and MR Egger slope estimates. Each point includes the 95% confidence intervals as error bars.
b. Funnel plot for MR spQRSTa-HCM X axis: Effect size of each variant. Y axis: Inverse of the variant's standard error. spQRSTa; Spatial QRS-T angle, HCM; Hypertrophic cardiomyopathy, MR; Mendelian Randomization.

a b

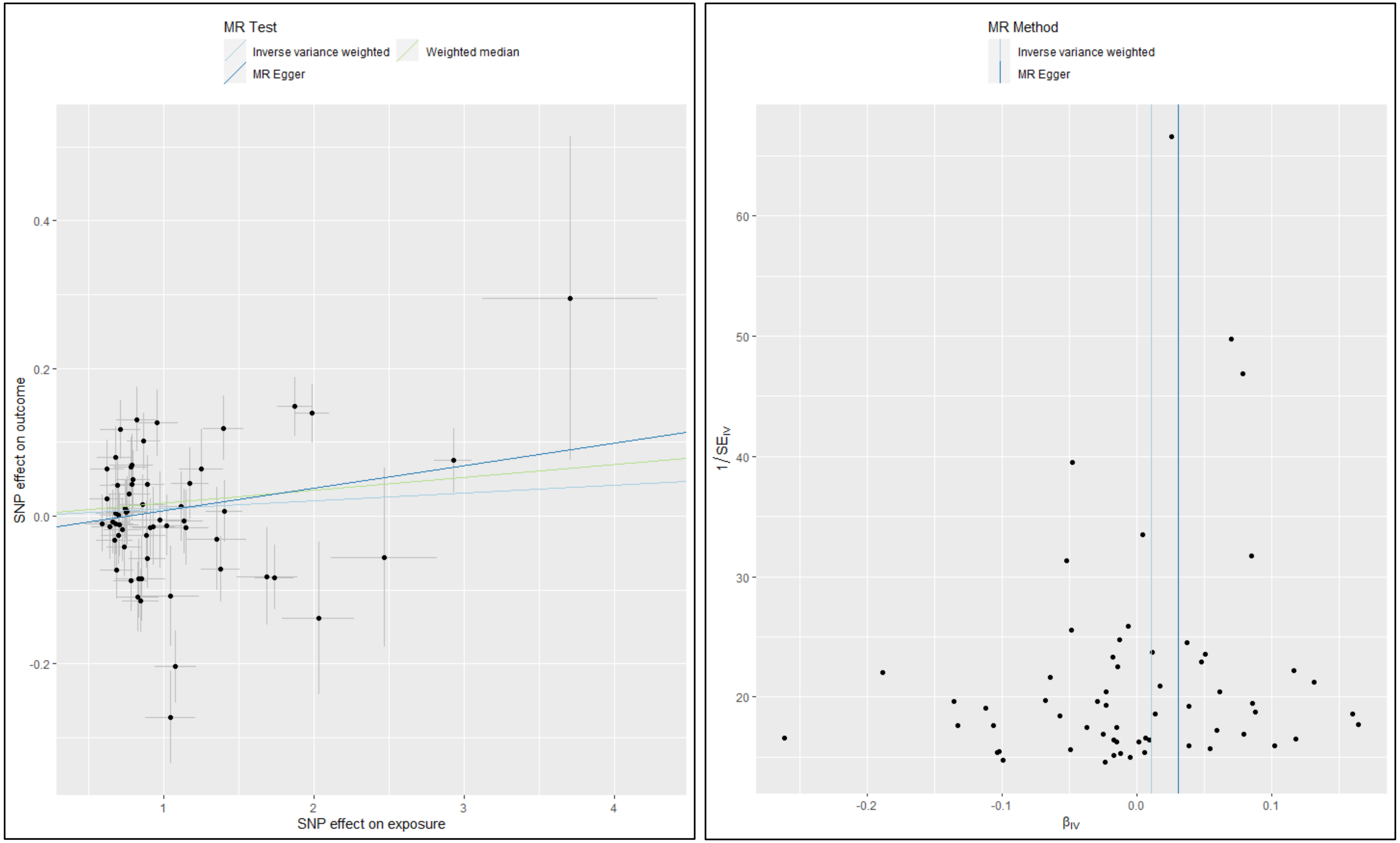

Supplementary Figure 9: Scatter and Funnel plots for spQRSTa-DCM multi-ancestry MR analyses.
a. Scatter plot of individual variant regression coefficients with Inverse-variance weighted and MR Egger slope estimates. Each point includes the 95% confidence intervals as error bars
b. Funnel plot for MR spQRSTa-DCM X axis: Effect size of each variant. Y axis: Inverse of the variant's standard error. spQRSTa; Spatial QRS-T angle, DCM; Dilated cardiomyopathy, MR; Mendelian Randomization.

Supplementary Notes:

Supplementary Note 1 – Study information for genome-wide association study meta-analysis

ARIC - Atherosclerosis Risk in Communities Study
The Atherosclerosis Risk in Communities (ARIC) Study is a prospective community-based study of cardiovascular disease and its risk factors. At baseline (1987-89), 15,792 men and women age 45-64 were recruited from 4 communities in the US (Washington County, Maryland; Forsyth County, North Carolina; Jackson, Mississippi; Minneapolis suburbs, Minnesota). Participants were mostly white in the Minnesota and Washington County field centers, white and African American in Forsyth County, and exclusively African American in the Jackson field center. Cohort members completed eight clinic examinations, conducted approximately three years apart between 1987 and 1998, with a fifth visit conducted from 2011 – 2013 and subsequent visits performed with a ninth currently in progress. Clinic examinations included assessment of cardiovascular risk factors, self-reported medical family history, employment and educational status, diet, physical activity, comorbidity, clinical and laboratory measurements. The present analyses utilized ECG measurements from the baseline assessment.

Digital 12-lead ECGs were obtained using MAC PC Personal Cardiographs (Marquette Electronics Inc., Milwaukee, WI) and were subsequently submitted to a central reading center at the EPICORE Center (University of Alberta, Edmonton, Alberta, Canada) and thereafter to the Epidemiological Cardiology Research Center (EPICARE), Wake Forest University, Winston-Salem, NC. All ECGs were visually inspected for quality and legibility at their acquisition and by the reading centers.

For assessment of QT interval, participants were asked not to smoke or ingest caffeine for at least 1 hour prior to the ECG being obtained. After resting for 5-10 minutes while the electrodes were being placed, a standard supine 12-lead electrocardiogram and a 2-minute paper recording of a three-lead (leads V1, II, and V5) rhythm strip were made. The QT interval from the digital 12-lead ECG was determined by identifying Q-onset and T-wave offset in all three leads. T-wave offset was defined as the point of maximum change of slope as the T-wave merges with the baseline as implemented in the Novacode ECG measurement and classification program as has been described in detail (https://pubmed.ncbi.nlm.nih.gov/9682893/) and used in prior ARIC studies of the QT interval (https://pubmed.ncbi.nlm.nih.gov/14975464/). U-waves were not detected by the Novacode algorithm. QRS and JT interval were measured automatically from baseline ECGs.

Bambui - Brazilian Bambuí Cohort Study of Ageing
A cohort study designed to identify predictors of adverse health events in the elderly. The study population comprises all residents of Bambuí (Minas Gerais, Brazil), aged 60 or more years (n=1.742) at the baseline in 1997. From these, 92.2% were interviewed and 85.9% underwent clinical examination, consisting of hematological and biochemical tests, serology for Trypanosoma cruzi, anthropometric and blood pressure measures and electrocardiogram. Cohort members undergo annual follow-up visits, which consist of an interview and verification of death certificates. Other procedures were repeated in selected years (2000, 2002 and 2008). From 1997 to 2007, during a mean follow-up of 8.6 years, 641 participants died and 96 (6.0%) were lost to follow-up.

BRIGHT - British Genetics of Hypertension
Participants of the BRIGHT Study were recruited from the Medical Research Council General Practice Framework and other primary care practices in the UK. Each case had a history of hypertension diagnosed prior to 60 years of age with confirmed blood pressure recordings corresponding to seated levels >150/100mmHg (1 reading) or mean of 3 readings >145/95 mmHg. BRIGHT is focused on recruitment of hypertensive individuals with BMI<30. Sample selection for GWAS was based on DNA availability and quantity.

CHRIS - The Cooperative Health Research in South Tyrol study
The Cooperative Health Research In South Tyrol (CHRIS) study is a population-based study with a longitudinal lookout to investigate the genetic and molecular basis of age-related common chronic

conditions and their interaction with life style and environment in the general population. The study was approved by the Ethics Committee of the Autonomous Province of Bolzano.

CHS - Cardiovascular Health Study
The Cardiovascular Health Study (CHS) is a population-based cohort study of risk factors for coronary heart disease and stroke in adults ≥65 years conducted across four field centers . The original predominantly European ancestry cohort of 5,201 persons was recruited in 1989-1990 from random samples of the Medicare eligibility lists; subsequently, an additional predominantly African-American cohort of 687 persons was enrolled in 1992-1993 for a total sample of 5,888.

Blood samples were drawn from all participants at their baseline examination and DNA was subsequently extracted from available samples. Genotyping was performed at the General Clinical Research Center's Phenotyping/Genotyping Laboratory at Cedars-Sinai among CHS participants who consented to genetic testing and had DNA available using the Illumina 370CNV BeadChip system (for European ancestry participants, in 2007) or the Illumina HumanOmni1-Quad_v1 BeadChip system (for African-American participants, in 2010).

CHS was approved by institutional review committees at each field center and individuals in the present analysis had available DNA and gave informed consent including consent to use of genetic information for the study of cardiovascular disease.

ERF - Erasmus Rucphen Family Study
The Erasmus Rucphen Family study is comprised of a family-based cohort embedded in the Genetic Research in Isolated Populations (GRIP) program in the southwest of the Netherlands. The aim of this program is to identify genetic risk factors for the development of complex disorders. In ERF, twenty-two families that had a large number of children baptized in the community church between 1850 and 1900 were identified with the help of detailed genealogical records. All living descendants of these couples, and their spouses, were invited to take part in the study. Comprehensive interviews, questionnaires, and examinations were completed at a research center in the area; approximately 3,200 individuals participated. Examinations included 12 lead ECG measurements. Electrocardiograms were recorded on ACTA electrocardiographs (ESAOTE, Florence, Italy) and digital measurements of the QRS and QT intervals were made using the Modular ECG Analysis System (MEANS). Data collection started in June 2002 and was completed in February 2005. In the current analyses, 2442 participants for whom complete phenotypic, genotypic and genealogical information was available were studied.

GAPP - Genetic and phenotypic determinants of blood pressure and other cardiovascular risk factors
GAPP is a population-based prospective cohort study involving a representative sample of healthy adults aged 25-41 years residing in the Principality of Liechtenstein. Exclusion criteria were the presence of cardiovascular disease, diabetes, obstructive sleep apnea and a body mass index >35kg/m2. A standardized 12-lead ECG was obtained in all participants.

GESUS - The Danish General Suburban Population Study
GESUS is a population-based prospective cohort study from Naestved Municipality (70km south of Copenhagen), Denmark. The study enrolled 21,205 adult participants between 2010-2013. Age was 20 years or above (20-100 years). All participants answered a questionnaire and had a physical examination (including EKG, laboratory tests, anthropometrics, biological samples for biobank etc.) performed at the Department of Clinical Biochemistry, Naestved Hospital, Denmark. A standard 12-lead paper ECG was obtained in all participants, and a corresponding electronic ECG was obtained in 8939 participants. ECG information was obtained from the MUSE Cardiology Information System (GE Healthcare, Wauwatosa, Wisconsin, USA) and analyzed by Marquette 12SL algorithm version 21. The ECGs were recorded with a sample rate of 500 Hz and a resolution of 4.88 μV per least significant bit. At the time of this study, 3004 participants were genotyped and included.

GS:SFHS - Generation Scotland: Scottish Family Health Study

The GS:SFHS study recruited 23,960 participants aged 18–100 years between 2006–11; full details are reported elsewhere [Smith et al, IJE 2012]. Participants came from across Scotland, with some family members from further afield. The sample was 59% female, with a wide range of ages and socio-demographic characteristics. Most (87%) participants were born in Scotland and 96% in the UK or Ireland.

HCHS/SOL - Hispanic Community Health Survey/Study of Latinos
The Hispanic Community Health Study/ Study of Latinos (HCHS/SOL) The HCHS/SOL is a multicenter, community-based cohort study of U.S. Hispanics/Latinos. Goals of the study are to examine the prevalence of and risk factors for several disorders including heart, lung, blood, and kidney phenotypes. HCHS/SOL investigators sampled 16,415 males and females aged 18-74 years at baseline from four study communities: The Bronx, NY, Chicago, IL, Miami, FL, and San Diego, CA. HCHS/SOL recruitment centers were selected so that the study would include at least 2,000 participants in each of the following designations: Mexican, Puerto Rican, Dominican, Cuban, and Central and South American.

INGI-FVG - INGI-Friuli Venezia Giulia
The INGI-FVG cohort consisted of about 1700 subjects drawn from the project "Genetic Park of Friuli Venezia Giulia". This study examined 6 isolated villages in the North-East of Italy between 2008 and 2010. Ethics approval was obtained from the Ethics Committee of the IRCCS Burlo Garofolo in Trieste. Written informed consent was obtained from every participant of the study. The study population had undergone clinical and instrumental evaluations. For all subjects, anthropometrics variables (such as height, weight, etc) were taken and a structured questionnaire about lifestyle and medical history was filled out. In addition, blood pressure, body-mass index, biochemical analyses, ECG and cardiovascular evaluation were collected.

INTER99 - Inter99
The Inter99 study carried out in 1999-2001 included invitation of12934 persons aged 30-60 years drawn from an age- and sex-stratified random sample of the population (16). The baseline participation rate was 52.5%, and the study included 6784 persons. The Inter99 study was a population-based randomized controlled trial (CT00289237, ClinicalTrials.gov) and investigated the effects of lifestyle intervention on CVD. Here 5827 participants with information on lipids and exome chip were analyzed. ECG information was obtained from the MUSE Cardiology Information System (GE Healthcare, Wauwatosa, Wisconsin) analyzed by Marquette 12SL algorithm version 21.

JHS - Jackson Heart Study
The JHS is a single-site cohort study of 5,306 extensively phenotyped African American women and men. Three clinical examinations have been completed, including the baseline examination, Examination 1 (2000–2004), Examination 2 (2005–2008), and Examination 3 (2009–2013), allowing comprehensive assessment of cardiovascular health and disease of the cohort at approximately four-year intervals. Ongoing monitoring of hospitalizations for cardiovascular events (coronary heart disease, heart failure and stroke) and deaths among cohort participants are accomplished by annual telephone follow-up interviews, surveillance of hospital discharge records (since 2000 for coronary heart disease and stroke, and since 2005 for heart failure), and vital records.

LIFELINES - LifeLines, a three-generation cohort study and biobank
The LifeLines Cohort Study, and generation and management of GWAS genotype data for the LifeLines Cohort Study is supported by the Netherlands Organization of Scientific Research NWO (grant 175.010.2007.006), the Economic Structure Enhancing Fund (FES) of the Dutch government, the Ministry of Economic Affairs, the Ministry of Education, Culture and Science, the Ministry for Health, Welfare and Sports, the Northern Netherlands Collaboration of Provinces (SNN), the Province of Groningen, University Medical Center Groningen, the University of Groningen, Dutch Kidney Foundation and Dutch Diabetes Research Foundation.

MESA - Multi-Ethnic Study of Atherosclerosis

The Multi-Ethnic Study of Atherosclerosis (MESA) is a study of the characteristics of subclinical cardiovascular disease (disease detected non-invasively before it has produced clinical signs and symptoms) and the risk factors that predict progression to clinically overt cardiovascular disease or progression of the subclinical disease. The cohort is a diverse, population-based sample of 6,814 asymptomatic men and women aged 45-84. Approximately 38 percent of the recruited participants are white, 28 percent African-American, 22 percent Hispanic, and 12 percent Asian (predominantly of Chinese descent). Participants were recruited during 2000-2002 from 6 field centers across the U.S. (at Wake Forest University; Columbia University; Johns Hopkins University; the University of Minnesota; Northwestern University; and the University of California – Los Angeles). All underwent anthropomorphic measurement and extensive evaluation by questionnaires at baseline, followed by 5 subsequent examinations at intervals of approximately 2-4 years. Age and sex were self-reported.

NEO - Netherlands Epidemiology of Obesity

The NEO was designed for extensive phenotyping to investigate pathways that lead to obesity-related diseases. The NEO study is a population-based, prospective cohort study that includes 6,671 individuals aged 45–65 years, with an oversampling of individuals with overweight or obesity. At baseline, information on demography, lifestyle, and medical history have been collected by questionnaires. In addition, samples of 24-h urine, fasting and postprandial blood plasma and serum, and DNA were collected. Genotyping was performed using the Illumina HumanCoreExome chip, which was subsequently imputed to the 1000 genome reference panel. Participants underwent an extensive physical examination, including anthropometry, electrocardiography, spirometry, and measurement of the carotid artery intima-media thickness by ultrasonography. In random subsamples of participants, magnetic resonance imaging of abdominal fat, pulse wave velocity of the aorta, heart, and brain, magnetic resonance spectroscopy of the liver, indirect calorimetry, dual energy X-ray absorptiometry, or accelerometry measurements were performed. The collection of data started in September 2008 and completed at the end of September 2012. Participants are currently being followed for the incidence of obesity-related diseases and mortality.

ORCADES - Orkney Complex Disease Study

The Orkney Complex Disease Study (ORCADES) is a family-based, cross-sectional study that seeks to identify genetic factors influencing cardiovascular and other disease risk in the isolated archipelago of the Orkney Isles in northern Scotland (McQuillan et al., 2008). Genetic diversity in this population is decreased compared to Mainland Scotland, consistent with the high levels of endogamy historically. 2078 participants aged 16-100 years were recruited between 2005 and 2011, most having three or four grandparents from Orkney, the remainder with two Orcadian grandparents. Fasting blood samples were collected and many health-related phenotypes and environmental exposures were measured in each individual. All participants gave written informed consent and the study was approved by Research Ethics Committees in Orkney and Aberdeen (North of Scotland REC).

PREVEND - Prevention of REnal and Vascular ENd stage Disease

Prevend is an ongoing prospective study investigating the natural course of increased levels of urinary albumin excretion and its relation to renal and cardiovascular disease. Details of the protocol have been described elsewhere.

PROSPER - PROSpective study of pravastatin in the elderly at Risk for vascular disease

All data come from the PROspective Study of Pravastatin in the Elderly at Risk (PROSPER). A detailed description of the study has been published elsewhere. PROSPER was a prospective multicenter randomized placebo-controlled trial to assess whether treatment with pravastatin diminishes the risk of major vascular events in elderly. Between December 1997 and May 1999, we screened and enrolled subjects in Scotland (Glasgow), Ireland (Cork), and the Netherlands (Leiden). Men and women aged 70-82 years were recruited if they had pre-existing vascular disease or increased risk of such disease because of smoking, hypertension, or diabetes. A total number of 5,804 subjects were randomly assigned to pravastatin or placebo. A large number of prospective tests were performed including Biobank tests and cognitive function measurements. A whole genome wide screening has been performed in the sequential PHASE project. Of 5,763 subjects DNA was available for genotyping.

Genotyping was performed with the Illumina 660K beadchip, after QC (call rate <95%) 5,244 subjects and 557,192 SNPs were left for analysis. These SNPs were imputed to 2.5 million SNPs based on the HAPMAP built 36 with MACH imputation software. The study was approved by the institutional ethics review boards of centers of Cork University (Ireland), Glasgow University (Scotland) and Leiden University Medical Center (the Netherlands) and all participants gave written informed consent.

RS - Rotterdam Study

Rotterdam Study, a prospective population-based cohort study among participants of European ancestry aged ≥ 40 years living in the well-defined Ommoord district of Rotterdam, the Netherlands. Details regarding design, objectives, and methods of the Rotterdam Study have been described in detail (Ikram et al, 2020) In short, in 1990, all inhabitants (n=10,215) aged 55 years or over were invited; 7,983 of invitees agreed to participate. In 2000, 3011 participants who had reached the age of 55 years (out of 4,472 invitees) were invited to participate in the second cohort. In 2006, a third cohort included inhabitants aged 45 years and older (n=3,932), bringing the total study population to 14,926 individuals by the end of 2008. There were no eligibility criteria to enter the Rotterdam Study apart from the minimum age and residential area based on postal codes. The Rotterdam Study has been approved by the Medical Ethics Committee of the Erasmus MC (registration number MEC 02.1015) and by the Dutch Ministry of Health, Welfare, and Sport (Population Screening Act WBO, license number 1071272-159521-PG). The Rotterdam Study entered into the Netherlands National Trial Register (NTR; www.trialregister.nl) and the WHO International Clinical Trials Registry Platform (ICTRP; www.who.int/ictrp/network/primary/en/) under shared catalog number NTR6831. 98% of participants provided written informed consent to participate in the study and obtain their information from treating physicians.

UK Biobank - UK Biobank Study

UK Biobank (UKB, www.ukbiobank.ac.uk) is a large longitudinal biobank study in the United Kingdom which was established to improve understanding of the genetic and environmental causes of common diseases including CVDs. In addition to self-reported disease outcomes and extensive health and life-style questionnaire data, UKB participants are being tracked through their NHS records and national registries (including cause of death and Hospital Episode Statistics). In 2017, UKB released the genotypes of 488,377 participants profiled with a custom SNP array. Genotyping QC was performed centrally by UKB, and genotypes imputed to Haplotype Reference Consortium (HRC) panel were released for 488,377 participants. For the UKB-12lead sub-cohort, ECG measures were calculated from a resting 12-lead ECG as previously described (Young et al, PMID 33537064).

VIKING - Viking Health Study

The Viking Health Study - Shetland (VIKING) is a family-based, cross-sectional study that seeks to identify genetic factors influencing cardiovascular and other disease risk in the population isolate of the Shetland Isles in northern Scotland. Genetic diversity in this population is decreased compared to Mainland Scotland, consistent with the high levels of endogamy historically. 2105 participants were recruited between 2013 and 2015, most having at least three grandparents from Shetland. Fasting blood samples were collected and many health-related phenotypes and environmental exposures were measured in each individual. All participants gave informed consent and the study was approved by the South East Scotland Research Ethics Committee.

WHI - Women's Health Initiative

The Women's Health Initiative (WHI) is a long-term national health study focused on strategies for preventing heart disease, breast and colorectal cancer, and osteoporotic fractures in postmenopausal women. Launched in 1993, the WHI enrolled 161,808 women aged 50-79 into one or more randomized Clinical Trials (CT), testing the health effects of hormone therapy (HT), dietary modification (DM), and/or calcium and Vitamin D supplementation (CaD) or to an Observational Study (OS). This ground-breaking study changed the way health care providers prevent and treat some of the major diseases impacting postmenopausal women. Results from the WHI Hormone Trials have been estimated to have already saved $35.2 billion in direct medical costs in the US alone. To date, WHI

has published over 1,400 articles and approved and funded 289 ancillary studies. The GWAS data used in this paper comes from six ancillary studies.

Supplementary Note 2 – Codes used to define each clinical outcome in the UK Biobank

ICD10 and ICD9 codes were used to extract hospital episode statistics and death registry information. OPCS4 are operation codes also used. The following codes were used to define atrial fibrillation, stroke, coronary artery disease, atrioventricular block/pacemaker implantation, bundle branch block/fascicular block, heart failure, non-ischaemic cardiomyopathy and ventricular arrhythmia.

**Atrial Fibrillation**	
ICD10 codes	Definition
I48	Atrial fibrillation
I48.0	Paroxysmal atrial fibrillation
I48.1	Persistent atrial fibrillation
I48.2	Chronic atrial fibrillation
ICD9 codes	Definition
4273	Atrial fibrillation and flutter
OPCS4	Definition
K62.1	Percutaneous transluminal ablation of pulmonary vein to left atrium conducting system
K62.5	Percutaneous transluminal occlusion of left atrial appendage
K57.1	Percutaneous transluminal ablation of atrioventricular node
K57.2	Percutaneous transluminal ablation of conducting system of heart NEC
**Stroke**	
ICD10 codes	Definition
I63.0	Cerebral infarction due to thrombosis of precerebral arteries
I63.1	Cerebral infarction due to embolism of precerebral arteries
I63.2	Cerebral infarction due to unspecified occlusion or stenosis of precerebral arteries
I63.3	Cerebral infarction due to thrombosis of cerebral arteries
I63.4	Cerebral infarction due to embolism of cerebral arteries
I63.5	Cerebral infarction due to unspecified occlusion or stenosis of cerebral arteries
I63.8	Other cerebral infarction
I63.9	Cerebral infarction, unspecified
I61.0	Intracerebral hemorrhage in hemisphere, subcortical
I61.1	Intracerebral hemorrhage in hemisphere, cortical
I61.2	Intracerebral hemorrhage in hemisphere, unspecified
I61.3	Intracerebral hemorrhage in brain stem
I61.4	Intracerebral hemorrhage in cerebellum
I61.5	Intracerebral hemorrhage, intraventricular
I61.6	Intracerebral hemorrhage, multiple localized
I61.8	Other intracerebral hemorrhage
I61.9	Intracerebral hemorrhage, unspecified
I64	Stroke, not specified as hemorrhage or infarction
ICD9 codes	Definition
434.91	Cerebral infarction due to thrombosis of precerebral arteries
434.91	Cerebral infarction due to embolism of precerebral arteries
434.91	Cerebral infarction due to unspecified occlusion or stenosis of precerebral arteries
434.01	Cerebral infarction due to thrombosis of cerebral arteries
434.11	Cerebral infarction due to embolism of cerebral arteries

434.91	Cerebral infarction due to unspecified occlusion or stenosis of cerebral arteries
434.91	Cerebral infarction, unspecified
436	Stroke, not specified as hemorrhage or infarction
**Coronary artery disease**	
ICD10 codes	Definition
I20	Unstable angina
I21.0	Acute transmural myocardial infarction of anterior wall
I21.1	Acute transmural myocardial infarction of inferior wall
I21.2	Acute transmural myocardial infarction of other sites
I21.3	Acute transmural myocardial infarction of unspecified site
I21.4	Acute subendocardial myocardial infarction
I21.9	Acute myocardial infarction, unspecified
I22.0	Subsequent myocardial infarction of anterior wall
I22.1	Subsequent myocardial infarction of inferior wall
I22.8	Subsequent myocardial infarction of other sites
I22.9	Subsequent myocardial infarction of unspecified site
I23.0	Hemopericardium as current complication following acute myocardial infarction
I23.1	Atrial septal defect as current complication following acute myocardial infarction
I23.2	Ventricular septal defect as current complication following acute myocardial infarction
I23.3	Rupture of cardiac wall without hemopericardium as current complication following acute myocardial infarction
I23.4	Rupture of chordae tendineae as current complication following acute myocardial infarction
I23.5	Rupture of papillary muscle as current complication following acute myocardial infarction
I23.6	Thrombosis of atrium , auricular appendage and ventricle as current complications following acute myocardial infarction
I23.8	Other current complications following acute myocardial infarction
I24.0	Coronary thrombosis not resulting in myocardial infarction
I24.8	Other forms of acute ischemic heart disease
I24.9	Acute ischemic heart disease, unspecified
I25.0	Atherosclerotic cardiovascular disease, so described
I25.1	Atherosclerotic heart disease
I25.2	Old myocardial infarction
I25.3	Aneurysm of heart
I25.4	Coronary artery aneurysm
I25.5	Ischemic cardiomyopathy
I25.6	Silent myocardial ischemia
I25.8	Other forms of chronic ischemic heart disease
I25.9	Chronic ischemic heart disease, unspecified
ICD9 codes	Definition
4109	Acute myocardial infarction
4119	Other acute and subacute forms of ischemic heart disease

4129	Old myocardial infarction
4139	Angina pectoris
4140	Coronary atherosclerosis
4141	Aneurysm of heart
4148	Other specified forms of chronic ischemic heart disease
4149	Chronic ischemic heart disease, unspecified
OPCS4	Definition
K40.1	Saphenous vein graft replacement of one coronary artery
K40.2	Saphenous vein graft replacement of two coronary arteries
K40.3	Saphenous vein graft replacement of three coronary arteries
K40.4	Saphenous vein graft replacement of four or more coronary arteries
K40.9	Unspecified saphenous vein graft replacement of coronary artery
K41.1	Autograft replacement of one coronary artery NEC
K41.2	Autograft replacement of two coronary arteries NEC
K41.3	Autograft replacement of three coronary arteries NEC
K41.4	Autograft replacement of four or more coronary arteries NEC
K42.4	Allograft replacement of four or more coronary arteries
K44.1	Replacement of coronary arteries using multiple methods
K44.2	Revision of replacement of coronary artery
K44.9	Unspecified other replacement of coronary artery
K45.1	Double anastomosis of mammary arteries to coronary arteries
K45.2	Double anastomosis of thoracic arteries to coronary arteries NEC
K45.3	Anastomosis of mammary artery to left anterior descending coronary artery
K45.4	Anastomosis of mammary artery to coronary artery NEC
K45.5	Anastomosis of thoracic artery to coronary artery NEC
K45.6	Revision of connection of thoracic artery to coronary artery
K45.8	Other specified connection of thoracic artery to coronary artery
K45.9	Unspecified connection of thoracic artery to coronary artery
K49.1	Percutaneous transluminal balloon angioplasty of one coronary artery
K49.2	Percutaneous transluminal balloon angioplasty of multiple coronary arteries
K49.3	Percutaneous transluminal balloon angioplasty of bypass graft of coronary artery
K49.4	Percutaneous transluminal cutting balloon angioplasty of coronary artery
K49.8	Other specified transluminal balloon angioplasty of coronary artery
K49.9	Unspecified transluminal balloon angioplasty of coronary artery
K50.1	Percutaneous transluminal laser coronary angioplasty
K50.2	Percutaneous transluminal coronary thrombolysis using streptokinase
K50.3	Percutaneous transluminal injection of therapeutic substance into coronary artery NEC
K50.4	Percutaneous transluminal atherectomy of coronary artery
K50.8	Other specified other therapeutic transluminal operations on coronary artery
K50.9	Unspecified other therapeutic transluminal operations on coronary artery
K75.1	Percutaneous transluminal balloon angioplasty and insertion of 1-2 drug-eluting stents into coronary artery
K75.2	Percutaneous transluminal balloon angioplasty and insertion of 3 or more drug-eluting stents into coronary artery

K75.3	Percutaneous transluminal balloon angioplasty and insertion of 1-2 stents into coronary artery
K75.4	Percutaneous transluminal balloon angioplasty and insertion of 3 or more stents into coronary artery NEC
K75.8	Other specified percutaneous transluminal balloon angioplasty and insertion of stent into coronary artery
K75.9	Unspecified percutaneous transluminal balloon angioplasty and insertion of stent into coronary artery
**Bundle branch block/fascicular block**	
ICD10 codes	Definition
I444	Left anterior fascicular block
I445	Left posterior fascicular block
I446	Other and unspecified fascicular block
I447	Left bundle-branch block, unspecified
ICD9 codes	Definition
4263	Other left bundle branch block
4264	Right bundle branch block
4265	Bundle branch block, unspecified
**Atrioventricular block**	
ICD10 codes	Definition
I440	Atrioventricular block, first degree
I441	Atrioventricular block, second degree
I442	Atrioventricular block, complete
I443	Other and unspecified atrioventricular block
ICD9 codes	Definition
4260	Atrioventricular block, complete
4261	Atrioventricular block, other and unspecified
**Heart Failure**	
ICD10 codes	Definition
I13.0	Hypertensive heart and renal disease with both (congestive) heart failure
I13.2	Hypertensive heart and renal disease with both (congestive) heart failure and renal failure
I50	Heart failure
I50.0	Congestive heart failure
I50.1	Left ventricular failure
I50.9	Heart failure, unspecified
ICD9 codes	Definition
4280	Congestive heart failure
4281	Left heart failure
4289	Heart failure, unspecified
OPCS4	Definition
K59.6	Implantation of cardioverter defibrillator using three electrode leads
K61.7	Implantation of biventricular cardiac pacemaker system

K60.7	Implantation of intravenous biventricular cardiac pacemaker system
**Non-ischaemic cardiomyopathy**	
ICD10 codes	Definition
I11.0	Hypertensive heart disease with (congestive) heart failure
I11.9	Hypertensive heart disease without (congestive) heart failure
I42.0	Dilated cardiomyopathy
I42.1	Obstructive hypertrophic cardiomyopathy
I42.2	Other hypertrophic cardiomyopathy
I42.3	Endomyocardial (eosinophilic) disease
I42.4	Endocardial fibroelastosis
I42.5	Other restrictive cardiomyopathy
I42.6	Alcoholic cardiomyopathy
I42.7	Cardiomyopathy due to drugs and other external agents
I42.8	Other cardiomyopathies
I42.9	Cardiomyopathy, unspecified
I43.0	Cardiomyopathy in infectious and parasitic diseases classified elsewhere
I43.1	Cardiomyopathy in metabolic diseases
I43.2	Cardiomyopathy in nutritional diseases
I43.8	Cardiomyopathy in other diseases classified elsewhere
ICD9 codes	Definition
4254	Dilated cardiomyopathy
42511	Obstructive hypertrophic cardiomyopathy
42518	Other hypertrophic cardiomyopathy
425	Endomyocardial (eosinophilic) disease
4253	Endocardial fibroelastosis
4254	Other restrictive cardiomyopathy
4255	Alcoholic cardiomyopathy
4259	Cardiomyopathy due to drugs and other external agents
4252/4254	Other cardiomyopathies
4254/4259	Cardiomyopathy, unspecified
4258	Cardiomyopathy in diseases classified elsewhere
4257	Cardiomyopathy in metabolic diseases
4257	Cardiomyopathy in nutritional diseases
4258	Cardiomyopathy in other diseases classified elsewhere
**Ventricular arrhythmia**	
ICD10 codes	Definition
I47.2	Ventricular tachycardia
I49.0	Ventricular fibrillation and flutter
ICD9 codes	Definition
4272	Paroxysmal ventricular tachycardia
4274	Ventricular fibrillation and flutter

Supplementary Note 3 – Study Acknowledgements

ARIC - Atherosclerosis Risk in Communities Study
The authors thank the staff and participants of the ARIC study for their important contributions.

BRIGHT - British Genetics of Hypertension
The BRIGHT study is extremely grateful to all the patients who participated in the study and the BRIGHT nursing team. This work forms part of the research program of the National Institutes of Health Research (NIHR), Barts Biomedical Centre award at Queen Mary University of London, UK.

CHRIS - The Cooperative Health Research in South Tyrol study
The CHRIS study is a collaborative effort between the Center for Biomedicine of the European Academy of Bolzano/Bozen (EURAC) and the Healthcare System of the Autonomous Province of Bolzano (Südtiroler Sanitätsbetrieb/Azienda Sanitaria dell'Alto Adige). The CHRIS Study is affiliated to the "German National Cohort" (Germany) and is indebted with the investigators of this study for their support in the study protocol definition. Full acknowledgements for the CHRIS study are reported here: http://translational-medicine.biomedcentral.com/articles/10.1186/s12967-015-0704-9#Declarations.

ERF - Erasmus Rucphen Family Study
We are grateful to all study participants and their relatives, general practitioners and neurologists for their contributions to the ERF study and to P Veraart for her help in genealogy, J Vergeer for the supervision of the laboratory work and P Snijders for his help in data collection.

GAPP - Genetic and phenotypic determinants of blood pressure and other cardiovascular risk factors
We thank the GAPP staff and all GAPP study participants for their important contributions

GESUS - The Danish General Suburban Population Study
We thank the participants and staff for their important contributions to science.

GS:SFHS - Generation Scotland: Scottish Family Health Study
We are grateful to all the families who took part, the general practitioners and the Scottish School of Primary Care for their help in recruiting them, and the whole Generation Scotland team, which includes interviewers, computer and laboratory technicians, clerical workers, research scientists, volunteers, managers, receptionists, healthcare assistants and nurses.

HCHS/SOL - Hispanic Community Health Survey/Study of Latinos
Hispanic Community Health Study/Study of Latinos (HCHS/SOL): We thank the participants and staff of the HCHS/SOL study for their contributions to this study. The baseline examination of HCHS/SOL was carried out as a collaborative study supported by contracts from the National Heart, Lung, and Blood Institute (NHLBI) to the University of North Carolina (N01-HC65233), University of Miami (N01-HC65234), Albert Einstein College of Medicine (N01-HC65235), Northwestern University (N01-HC65236) and San Diego State University (N01-HC65237).

INGI-FVG - INGI- Friuli Venezia Giulia
We are very grateful to the municipal administrators for their collaboration on the project and for logistic support. We would like to thank all participants to this study.

INTER99 - Inter99
The Inter99 was initiated by Torben Jørgensen (PI), Knut Borch-Johnsen (co-PI), Hans Ibsen and Troels F. Thomsen. The steering committee comprises the former two and Charlotta Pisinger.

JHS - Jackson Heart Study
We thank the Jackson Heart Study (JHS) participants and staff for their contributions to this work.

LIFELINES - LifeLines, a three-generation cohort study and biobank
We thank U. Bultmann (1), J.M. Geleijnse (2), P. van der Harst (3), S. Mulder (4), J.G.M. Rosmalen (5), E.F.C. van Rossum (6), H.A. Smit (7), M. Swertz (8), E.A.L.M. Verhagen (9).
(1) Department of Social Medicine, University Medical Center Groningen, University of Groningen, The Netherlands (2) Department of Human Nutrition, Wageningen University, The Netherlands (3) Department of Cardiology, University Medical Center Groningen, University of Groningen, The Netherlands (4) Lifelines Cohort Study, The Netherlands (5) Interdisciplinary Center of Psychopathology of Emotion Regulation (ICPE), Department of Psychiatry, University Medical Center Groningen, University of Groningen, The Netherlands (6) Department of Endocrinology, Erasmus Medical Center, Rotterdam, The Netherlands (7) Department of Public Health, University Medical Center Utrecht, The Netherlands (8) Department of Genetics, University Medical Center Groningen, University of Groningen, The Netherlands (9) Department of Public and Occupational Health, VU Medical Center, Amsterdam, The Netherlands

MESA - Multi-Ethnic Study of Atherosclerosis
The authors thank the MESA participants and the MESA investigators and staff for their valuable contributions. A full list of participating MESA investigators and institutions can be found at http://www.mesa-nhlbi.org.

NEO - Netherlands Epidemiology of Obesity
The authors of the NEO study thank all individuals who participated in the Netherlands Epidemiology in Obesity study, all participating general practitioners for inviting eligible participants and all research nurses for collection of the data. We thank the NEO study group, Pat van Beelen, Petra Noordijk and Ingeborg de Jonge for the coordination, lab and data management of the NEO study. We also thank Arie Maan for the analyses of the electrocardiograms.

ORCADES - Orkney Complex Disease Study
DNA extractions were performed at the Wellcome Trust Clinical Research Facility in Edinburgh. We would like to acknowledge the invaluable contributions of the research nurses in Orkney, the administrative team in Edinburgh and the people of Orkney.

PROSPER - PROSpective study of pravastatin in the elderly at Risk for vascular disease
The PROSPER study was supported by an investigator initiated grant obtained from Bristol-Myers Squibb. J.Wouter Jukema is an Established Clinical Investigator of the Netherlands Heart Foundation (grant 2001 D 032). Support for genotyping was provided by the seventh framework program of the European commission (grant 223004) and by the Netherlands Genomics Initiative (Netherlands Consortium for Healthy Aging grant 050-060-810).

RS - Rotterdam Study
We thank Pascal Arp, Mila Jhamai, Dr Michael Moorhouse, Marijn Verkerk, and Sander Bervoets for their help in creating the GWAS database. The authors are very grateful to the participants and staff from the Rotterdam Study, the participating general practitioners and the pharmacists.

UK Biobank - UK Biobank Study
This research has been conducted using the UK Biobank Resource (application 8256 - Understanding genetic influences in the response of the cardiac electrical system to exercise). This work forms part of the research program of the National Institutes of Health Research (NIHR) Barts Biomedical Centre, Queen Mary University of London, UK. PD Lambiase acknowledges support from the UCLH Biomedicine NIHR. JR acknowledges support from the Marie Sklodowska-Curie grant No 786833 and from the "María Zambrano" fellowship through the European Union-NextGenerationEU.

VIKING - Viking Health Study

DNA extractions and genotyping were performed at the Edinburgh Clinical Research Facility, University of Edinburgh. We would like to acknowledge the invaluable contributions of the research nurses in Shetland, the administrative team in Edinburgh and the people of Shetland.

WHI - Women's Health Initiative
The WHI program is supported by contracts from the National Heart, Lung and Blood Institute, NIH. The authors thank the WHI investigators and staff for their dedication, and the study participants for making the program possible. A listing of WHI investigators can be found at http://www.whi.org/researchers/Documents%20%20Write%20a%20Paper/WHI%20Investigator%20 Short%20List.pdf. Scientific Computing Infrastructure at the Fred Hutchinson Cancer Center is funded by ORIP grant S10OD028685.

Additional acknowledgements
This research utilized Queen Mary's Apocrita HPC facility, supported by QMUL Research-IT. http://doi.org/10.5281/zenodo.438045

Supplementary Note 4 – Study Funding

ARIC - Atherosclerosis Risk in Communities Study
The Atherosclerosis Risk in Communities study has been funded in whole or in part with Federal funds from the National Heart, Lung, and Blood Institute, National Institutes of Health, Department of Health and Human Services, under Contract nos. (HHSN268201700001I, HHSN268201700002I, HHSN268201700003I, HHSN268201700004I, HHSN268201700005I). Funding was also supported by R01HL087641, R01HL059367 and R01HL086694; National Human Genome Research Institute contract U01HG004402; and National Institutes of Health contract HHSN268200625226C. Infrastructure was partly supported by Grant Number UL1RR025005, a component of the National Institutes of Health and NIH Roadmap for Medical Research.

Bambui - Brazilian Bambuí Cohort Study of Ageing
Brazilian Ministry of Health (DECIT/MS, EPIGEN-Brazil Project), Brazilian Ministry of Science and Technology (Financiadora de Estudos e Projetos (FINEP), Brazilian Conselho Nacional de Desenvolvimento Científico e Tecnologico (CNPq), Fundação de Amparo a Pesquisa do Estado de Minas Gerais (FAPEMIG).

BRIGHT - British Genetics of Hypertension
This work was supported by the Medical Research Council (MRC) of Great Britain (grant number G9521010D) and the British Heart Foundation (grant number PG/02/128).

CHRIS - The Cooperative Health Research in South Tyrol study
The CHRIS study was funded by the Department of Innovation, Research, and University of the Autonomous Province of Bolzano-South Tyrol.

CHS - Cardiovascular Health Study
Cardiovascular Health Study: This CHS research was supported by NHLBI contracts HHSN268200960009C, HHSN268201200036C, HHSN268200800007C, HHSN268201800001C, N01HC55222, N01HC85079, N01HC85080, N01HC85081, N01HC85082, N01HC85083, N01HC85086, 75N92021D00006; and NHLBI grants U01HL080295, R01HL085251, R01HL087652, R01HL105756, R01HL103612, R01HL120393, and U01HL130114 with additional contribution from the National Institute of Neurological Disorders and Stroke (NINDS). Additional support was provided through R01AG023629 from the National Institute on Aging (NIA). A full list of principal CHS investigators and institutions can be found at CHS-NHLBI.org. The provision of genotyping data was supported in part by the National Center for Advancing Translational Sciences, CTSI grant UL1TR001881, and the National Institute of Diabetes and Digestive and Kidney Disease Diabetes Research Center (DRC) grant DK063491 to the Southern California Diabetes Endocrinology Research Center. The content is solely the responsibility of the authors and does not necessarily represent the official views of the National Institutes of Health.

ERF - Erasmus Rucphen Family Study
The ERF study as a part of EUROSPAN (European Special Populations Research Network) was supported by European Commission FP6 STRP grant number 018947 (LSHG-CT-2006-01947) and also received funding from the European Community's Seventh Framework Programme (FP7/2007-2013)/grant agreement HEALTH-F4-2007-201413 by the European Commission under the programme “Quality of Life and Management of the Living Resources” of 5th Framework Programme (no. QLG2-CT-2002-01254). The ERF study was further supported by ENGAGE consortium and CMSB. High-throughput analysis of the ERF data was supported by joint grant from Netherlands Organisation for Scientific Research and the Russian Foundation for Basic Research (NWO-RFBR 047.017.043).

GAPP - Genetic and phenotypic determinants of blood pressure and other cardiovascular risk factors

The GAPP study was supported by the Liechtenstein Government, the Swiss Heart Foundation, the Swiss Society of Hypertension, the University of Basel, the University Hospital Basel, the Hanela Foundation, the Mach-Gaensslen Foundation, Schiller AG, and Novartis.

Garnier et al – DCM GWAS meta-analysis
This research was supported by the Laboratory of Excellence GENMED (Medical Genomics) grant no. ANR-10-LABX-0013 managed by the National Research Agency (ANR) part of the Investment for the Future program.

GESUS - The Danish General Suburban Population Study
GESUS was funded by the Region Zealand Research Foundation, Naestved Hospital Foundation, Naestved Municipality, Johan and Lise Boserup Foundation, TrygFonden, Johannes Fog's Foundation, Region Zealand, Naestved Hospital, The National Board of Health, The Local Government Denmark Foundation. None of the funding sources had any influence on any part of this study or manuscript. Genotyping was funded by The Hallas-Møller Emerging Investigator Novo Nordisk (NNF17OC0031204).

GS:SFHS - Generation Scotland: Scottish Family Health Study
Generation Scotland received core support from the Chief Scientist Office of the Scottish Government Health Directorates [CZD/16/6] and the Scottish Funding Council [HR03006]. Genotyping of the GS:SFHS samples was carried out by the Genetics Core Laboratory at the t Clinical Research Facility, University of Edinburgh, Scotland and was funded by the Medical Research Council UK and the Wellcome Trust (Wellcome Trust Strategic Award "STratifying Resilience and Depression Longitudinally" (STRADL) Reference 104036/Z/14/Z).

HCHS/SOL - Hispanic Community Health Survey/Study of Latinos
This work was supported by funding from the National Heart, Lunch and Blood Institute (N01-HC65233, N01-HC65234, N01-HC65235, N01-HC65236, N01-HC65237, and T32HL7055). Christy

INGI-FVG - INGI-Friuli Venezia Giulia
Italian Ministry of Health - RC 35/17; Italian Ministry of Education, University and Research, D70-RESRICGIROTTO to GG

INTER99 - Inter99
The study was financially supported by research grants from the Danish Research Council, the Danish Centre for Health Technology Assessment, Novo Nordisk Inc., Research Foundation of Copenhagen County, Ministry of Internal Affairs and Health, the Danish Heart Foundation, the Danish Pharmaceutical Association, the Augustinus Foundation, the Ib Henriksen Foundation, the Becket Foundation, and the Danish Diabetes Association.

JHS - Jackson Heart Study
The Jackson Heart Study (JHS) is supported and conducted in collaboration with Jackson State University (HHSN268201800013I), Tougaloo College (HHSN268201800014I), the Mississippi State Department of Health (HHSN268201800015I/HHSN26800001) and the University of Mississippi Medical Center (HHSN268201800010I, HHSN268201800011I and HHSN268201800012I) contracts from the National Heart, Lung, and Blood Institute (NHLBI) and the National Institute for Minority Health and Health Disparities (NIMHD). The authors also wish to thank the staffs and participants of the JHS.

LIFELINES - LifeLines, a three-generation cohort study and biobank
The LifeLines Cohort Study, and generation and management of GWAS genotype data for the LifeLines Cohort Study is supported by the Netherlands Organization of Scientific Research NWO (grant 175.010.2007.006), the Economic Structure Enhancing Fund (FES) of the Dutch government, the Ministry of Economic Affairs, the Ministry of Education, Culture and Science, the Ministry for Health, Welfare and Sports, the Northern Netherlands Collaboration of Provinces (SNN), the Province

of Groningen, University Medical Center Groningen, the University of Groningen, Dutch Kidney Foundation and Dutch Diabetes Research Foundation.

MESA - Multi-Ethnic Study of Atherosclerosis
MESA and the MESA SHARe project are conducted and supported by the National Heart, Lung, and Blood Institute (NHLBI) in collaboration with MESA investigators. Support for MESA is provided by contracts 75N92020D00001, HHSN268201500003I, N01-HC-95159, 75N92020D00005, N01-HC-95160, 75N92020D00002, N01-HC-95161, 75N92020D00003, N01-HC-95162, 75N92020D00006, N01-HC-95163, 75N92020D00004, N01-HC-95164, 75N92020D00007, N01-HC-95165, N01-HC-95166, N01-HC-95167, N01-HC-95168, N01-HC-95169, UL1-TR-000040, UL1-TR-001079, UL1-TR-001420, UL1-TR-001881, and DK063491.

NEO - Netherlands Epidemiology of Obesity
The genotyping in the NEO study was supported by the Centre National de Génotypage (Paris, France), headed by Jean-Francois Deleuze. The NEO study is supported by the participating Departments, the Division and the Board of Directors of the Leiden University Medical Center, and by the Leiden University, Research Profile Area Vascular and Regenerative Medicine.

ORCADES - Orkney Complex Disease Study
The Orkney Complex Disease Study (ORCADES) was supported by the Chief Scientist Office of the Scottish Government (CZB/4/276, CZB/4/710), a Royal Society URF to James F Wilson, the MRC Human Genetics Unit quinquennial programme "QTL in Health and Disease", Arthritis Research UK and the European Union framework program 6 EUROSPAN project (contract no. LSHG-CT-2006-018947).

PREVEND - Prevention of REnal and Vascular ENd stage Disease
PREVEND genetics is supported by the Dutch Kidney Foundation (Grant E033), the EU project grant GENECURE (FP-6 LSHM CT 2006 037697), the National Institutes of Health (grant 2R01LM010098), The Netherlands organization for health research and development (NWO-Groot grant 175.010.2007.006, NWO VENI grant 916.761.70, ZonMw grant 90.700.441), and the Dutch Inter University Cardiology Institute Netherlands (ICIN). N Verweij was supported by NWO VENI (016.186.125).

PROSPER - PROSpective study of pravastatin in the elderly at Risk for vascular disease
The PROSPER study was supported by an investigator initiated grant obtained from Bristol-Myers Squibb. J.Wouter Jukema is an Established Clinical Investigator of the Netherlands Heart Foundation (grant 2001 D 032). Support for genotyping was provided by the seventh framework program of the European commission (grant 223004) and by the Netherlands Genomics Initiative (Netherlands Consortium for Healthy Aging grant 050-060-810).

RS - Rotterdam Study
The Rotterdam Study is supported by the Erasmus Medical Center and Erasmus University Rotterdam; the Netherlands Organization for Scientific Research (NWO); the Netherlands Organization for Health Research and Development (ZonMw); the Research Institute for Diseases in the Elderly (RIDE); the Netherlands Heart Foundation; the Ministry of Education, Culture and Science; the Ministry of Health Welfare and Sports; the European Commission; and the Municipality of Rotterdam. Support for genotyping was provided by the Netherlands Organisation of Scientific Research NWO Investments (nr. 175.010.2005.011, 911-03-012), the Research Institute for Diseases in the Elderly (014-93-015; RIDE2), the Netherlands Genomics Initiative (NGI)/Netherlands Consortium for Healthy Aging (NCHA) project nr. 050- 060-810. The GWA study was funded by the Netherlands Organisation of Scientific Research NWO Investments (nr. 175.010.2005.011, 911-03-012), the Research Institute for Diseases in the Elderly (014-93-015; RIDE2), the Netherlands Genomics Initiative (NGI)/Netherlands Consortium for Healthy Aging (NCHA) project nr. 050-060-810.

UK Biobank - UK Biobank Study

No study funding to declare.

VIKING - Viking Health Study
The Viking Health Study – Shetland (VIKING) was supported by the MRC Human Genetics Unit quinquennial programme grant “QTL in Health and Disease”.

WHI - Women's Health Initiative
The WHI program is funded by the National Heart, Lung, and Blood Institute, National Institutes of Health, U.S. Department of Health and Human Services through contracts 75N92021D00001, 75N92021D00002, 75N92021D00003, 75N92021D00004, 75N92021D00005. The Modification of PM-Mediated Arrhythmogenesis in Populations (WHI-MOPMAP) was funded by the NIEHS (R01-ES017794, Whitsel).
