# Supplementary material for: Genetic architecture of spatial electrical biomarkers for cardiac arrhythmia and relationship with cardiovascular disease
Source: Nat Commun. 2023 Mar 14;14:1411. doi: 10.1038/s41467-023-36997-w (PMC10015012; doi:10.1038/s41467-023-36997-w)
Supplement: Supplementary file 3 — Description of Additional Supplementary Files [file 41467_2023_36997_MOESM3_ESM.pdf]

Description of Additional Supplementary Files

File Name: Supplementary Data 1
Description: Study information for all participating studies

File Name: Supplementary Data 2
Description: Per study (and their sub-studies) summary of genotyping and GWAS software information

File Name: Supplementary Data 3
Description: Per study summary statistics of ECG measures and covariates

File Name: Supplementary Data 4
Description: Lead variants from independent loci for the spatial QRS-T angle

File Name: Supplementary Data 5
Description: Lead variants from independent loci for the frontal QRS-T angle

File Name: Supplementary Data 6
Description: Conditionally independent variants for spatial and frontal QRS-T angle in individuals of European ancestry from UK Biobank

File Name: Supplementary Data 7
Description: Annotation of lead variants and proxies for spatial QRS-T angle loci from multi-ancestry meta-analyses.

File Name: Supplementary Data 8
Description: Annotation of variants with Combined Annotation Dependent Depletion (CADD) scores.

File Name: Supplementary Data 9
Description: Significant cis-eQTLs in cardiac and vascular tissues.

File Name: Supplementary Data 10
Description: Association of gene expression with spQRSTa and fQRSTa using S-PrediXcan

File Name: Supplementary Data 11a
Description: Hi-C results for lead variants and proxies ($r2 > 0.8$) for each multi-ancestry meta-analysis using Fit-Hi-C pipeline

File Name: Supplementary Data 11b
Description: Promoter-capture Hi-C results for lead variants and proxies ($r2 > 0.8$) from each multi-ancestry meta-analysis.

File Name: Supplementary Data 12
Description: Gene-set tissue/cell type encrichment using Data driven Expression-Prioritisation

Integration for Complex Traits (DEPICT) for spatial QRS-T angle multi-ancestry meta-analysis.

File Name: Supplementary Data 13
Description: DEPICT pathway geneset enrichment results for spQRSTa multi-ancestry meta-analysis.

File Name: Supplementary Data 14
Description: Summary of bioinformatic findings and potential candidate genes at each locus for spQRSTa multi-ancestry loci

File Name: Supplementary Data 15
Description: Overlap of spQRSTa loci with other resting ECG measures

File Name: Supplementary Data 16
Description: Look up of lead variants and their proxies from each multi-ancestry meta-analysis in Phenoscanner for overlap with other phenotypes in GWAS

File Name: Supplementary Data 17
Description: Association of genetically determined spQRSTa and fQRSTa with cardiovascular disease in UK Biobank

File Name: Supplementary Data 18
Description: Two sample Mendelian Randomisation for spQRSTa and HCM

File Name: Supplementary Data 19
Description: Two sample Mendelian Randomisation for spQRSTa and DCM
