# Supplementary material for: Genetic architecture of spatial electrical biomarkers for cardiac arrhythmia and relationship with cardiovascular disease
Source: Nat Commun. 2023 Mar 14;14:1411. doi: 10.1038/s41467-023-36997-w (PMC10015012; doi:10.1038/s41467-023-36997-w)
Supplement: Supplementary file 5 — Reporting Summary [file 41467_2023_36997_MOESM5_ESM.pdf]

Corresponding author(s): Patricia Munroe and Larisa Tereshchenko

Last updated by author(s): 19th December 2022

Reporting Summary

Nature Portfolio wishes to improve the reproducibility of the work that we publish. This form provides structure for consistency and transparency in reporting. For further information on Nature Portfolio policies, see our Editorial Policies and the Editorial Policy Checklist.

Statistics

For all statistical analyses, confirm that the following items are present in the figure legend, table legend, main text, or Methods section.

n/a	Confirmed	
☐	☒	The exact sample size (*n*) for each experimental group/condition, given as a discrete number and unit of measurement
☐	☒	A statement on whether measurements were taken from distinct samples or whether the same sample was measured repeatedly
☐	☒	The statistical test(s) used AND whether they are one- or two-sided<br>*Only common tests should be described solely by name; describe more complex techniques in the Methods section.*
☐	☒	A description of all covariates tested
☐	☒	A description of any assumptions or corrections, such as tests of normality and adjustment for multiple comparisons
☐	☒	A full description of the statistical parameters including central tendency (e.g. means) or other basic estimates (e.g. regression coefficient) AND variation (e.g. standard deviation) or associated estimates of uncertainty (e.g. confidence intervals)
☐	☒	For null hypothesis testing, the test statistic (e.g. *F*, *t*, *r*) with confidence intervals, effect sizes, degrees of freedom and *P* value noted<br>*Give P values as exact values whenever suitable.*
☐	☒	For Bayesian analysis, information on the choice of priors and Markov chain Monte Carlo settings
☒	☐	For hierarchical and complex designs, identification of the appropriate level for tests and full reporting of outcomes
☐	☒	Estimates of effect sizes (e.g. Cohen's *d*, Pearson's *r*), indicating how they were calculated

Our web collection on statistics for biologists contains articles on many of the points above.

Software and code

Policy information about availability of computer code

Data collection	No software was used for data collection centrally. Study level summary statistics were transferred for central analysis using the Globus file-transfer platform (https://www.globus.org/)
Data analysis	METAL: https://genome.sph.umich.edu/wiki/METAL_Documentation, version released 2011-03-25<br>Genome-wide Complex Trait Analysis (GCTA, v1.26.0): https://cnsgenomics.com/software/gcta/#Overview<br>R version 3.5.3: https://cran.r-project.org/<br>LD SCore software (LDSC, v1.0.1): https://github.com/bulik/ldsc/wiki/Heritability-and-Genetic-Correlation<br>BOLT-Restricted Maximum Likelihood (REML, v2.3.2): https://alkesgroup.broadinstitute.org/BOLT-LMM/BOLT-LMM_manual.html<br>Variant Effect Predictor (VEP), Ensembl release 99: https://www.ensembl.org/info/docs/tools/vep/index.html<br>Combined Annotation Dependent Depletion (CADD, v1.4): https://cadd.gs.washington.edu/<br>GTEx version 8: https://gtexportal.org/home/<br>Functional Mapping and Annotation of Genome-Wide Association Studies (FUMA GWAS, v1.3.6): https://fuma.ctglab.nl/<br>CHEERS (version accessed 2020): https://github.com/TrynkaLab/CHEERS<br>GWAS Analysis of Regulatory and Functional Information Enrichment with LD correction (GARFIELD, v2): https://www.ebi.ac.uk/birney-srv/GARFIELD/<br>Data-driven Expression-Prioritization Integration for Complex Traits (DEPICT, v3): https://github.com/perslab/depict<br>PRSice-2: https://www.prsice.info/<br>R colocalization package (COLOC, v 5.1.0): https://cran.r-project.org/web/packages/coloc/index.html<br>EasyQC R package (v9.2): https://www.uni-regensburg.de/medizin/epidemiologie-praeventivmedizin/genetische-epidemiologie/software/index.html<br>Locuszoom: http://locuszoom.org/

RegulomeDB (v.2.0.3): https://regulomedb.org/regulome-search/
PLINK v1.90: https://www.cog-genomics.org/plink/
Liftover tool (version July 2015): https://genome.sph.umich.edu/wiki/LiftOver
S-PrediXcan (v0.6.11): https://github.com/hakyimlab/MetaXcan
REVIGO (accessed September 2019): http://revigo.irb.hr/
Cytoscape (v3.8.2): https://cytoscape.org/
GWAS-PW v0.21: https://github.com/joepickrell/gwas-pw
PheWAS R package (v0.99.5-5): https://github.com/PheWAS/PheWAS
Phenoscanner v2: http://www.phenoscanner.medschl.cam.ac.uk/
TwoSampleMR R package (v0.5.6): https://mrcieu.github.io/TwoSampleMR/
Sorting Intolerant From Tolerant algorithm (SIFT, version 5.2.2): https://www.ensembl.org/info/docs/tools/vep/index.html, https://sift.bii.a-star.edu.sg/
PolyPhen-2 (v2.2.2): https://www.ensembl.org/info/docs/tools/vep/index.html, http://genetics.bwh.harvard.edu/pph2/

For manuscripts utilizing custom algorithms or software that are central to the research but not yet described in published literature, software must be made available to editors and reviewers. We strongly encourage code deposition in a community repository (e.g. GitHub). See the Nature Portfolio guidelines for submitting code & software for further information.

Data

Policy information about availability of data

All manuscripts must include a data availability statement. This statement should provide the following information, where applicable:

- Accession codes, unique identifiers, or web links for publicly available datasets
- A description of any restrictions on data availability
- For clinical datasets or third party data, please ensure that the statement adheres to our policy

Summary statistics from each genome-wide association study meta-analysis will be made available on the NHGRI-EBI Catalog of human genome-wide association studies website, https://www.ebi.ac.uk/gwas/.

Data relating to UK Biobank will be return to the study. The UK Biobank will make these data available to all bona fide researchers for all types of health-related research that is in the public interest, without preferential or exclusive access for any person. All researchers will be subject to the same application process and approval criteria as specified by the UK Biobank. Please see the UK Biobank's website for the detailed access procedure (http:/www.ukbiobank.ac.uk/register-apply/).

Other datasets used in these analyses are publically available and can be sourced from:
1000 Genomes reference panel (NCBI build 37): https://www.internationalgenome.org/category/reference/
Haplotype reference consortium reference panel (NCBI build 37): http://www.haplotype-reference-consortium.org/
Variant level annotation from Variant Effect Predictor (VEP), Ensembl release 99: https://www.ensembl.org/info/docs/tools/vep/index.html
Variant level Combined Annotation Dependent Depletion scores from Combined Annotation Dependent Depletion (CADD, v1.4): https://cadd.gs.washington.edu/
Variant level tissue-specific gene expression from The GTEx portal (v8): https://gtexportal.org/home/
HiC data from the Functional Mapping and Annotation of Genome-Wide Association Studies (FUMA GWAS, v1.3.6): https://fuma.ctglab.nl/
DNaseI hypersensivity site enrichment data from GWAS Analysis of Regulatory and Functional Information Enrichment with LD correction (GARFIELD, v2): https://www.ebi.ac.uk/birney-srv/GARFIELD/
Gene-set, biological pathways and tissue expression data from Data-driven Expression-Prioritization Integration for Complex Traits (DEPICT, v3): https://github.com/perslab/depict
Variant level RegulomeDB scores from RegulomeDB (v.2.0.3): https://regulomedb.org/regulome-search/
A compendium of promoter-centered long-range chromatin interactions in the human genome (Jung et al, 2019): https://doi.org/10.1038/s41588-019-0494-8
Cardiac cell type-specific gene regulatory programs and disease risk association (Hocker et al, 2021): DOI: 10.1126/sciadv.abf1444
Druggable genome dataset from Finan et al, 2017: DOI: 10.1126/scitranslmed.aag1166
g:Profiler (accessed May 2021): https://biit.cs.ut.ee/gprofiler/gost
Online Mendelian Inheritance in Man database (accessed September 2021): https://www.omim.org/
Mouse Genome Informatics (accessed September 2021): http://www.informatics.jax.org/

Human research participants

Policy information about studies involving human research participants and Sex and Gender in Research.

Reporting on sex and gender	Sex-stratified analyses have not been performed in this study. Therefore these findings do not apply to one sex.
Population characteristics	These analyses included 23 studies and their sub-studies. These studies included population samples and controls from case-control studies. Supplementary tables contain detailed information for each study including study design and descriptive statistics.
Recruitment	Studies were recruited to this meta-analysis from the CHARGE consortium, an international genetics consortium. Studies participated if they had spQRSTa and fQRSTa ECG measures, genetic data and the required covariates. An analysis plan was created and shared with all studies to harmonise the approach (methods).
Ethics oversight	All participating studies approved of this project. Ethics was obtained at a study level and and informed consent was obtained from all participants.

Note that full information on the approval of the study protocol must also be provided in the manuscript.

Field-specific reporting

Please select the one below that is the best fit for your research. If you are not sure, read the appropriate sections before making your selection.

☒ Life sciences ☐ Behavioural & social sciences ☐ Ecological, evolutionary & environmental sciences

For a reference copy of the document with all sections, see nature.com/documents/nr-reporting-summary-flat.pdf

Life sciences study design

All studies must disclose on these points even when the disclosure is negative.

Sample size	This study was performed with the aim to facilitate discovery of new loci for the spQRSTa and fQRSTa. Therefore the largest sample size possible was used and power calculations were not performed
Data exclusions	Individuals were excluded at the study level for: prevalent myocardial infarction or heart failure, pregnancy at the time of recruitment, implantation of a pacemaker or implantable cardiac defibrillator, QRS duration greater than 120ms, or right or left bundle branch block or atrial fibrillation on ECG. Additionally, if the data was available, individuals using digitalis, class I or III anti-arrhythmics or QT prolonging medication were excluded.<br>An imputation quality cut-off of Rsq > 0.3 (or similar in IMPUTE) and minor allele frequency >0.05 was applied in all cohorts to ensure high quality variants were included in the meta-analysis. Variants with invalid beta estimates, standard errors or P-values were removed. Further information is available in the methods section.
Replication	A discovery analysis only was performed due to the lack of a suitable sized replication dataset.
Randomization	Randomization is not necessary to perform genome-wide association studies and was not applicable for this study.
Blinding	Blinding is not necessary to perform genome-wide association studies and was not applicable for this study.

Reporting for specific materials, systems and methods

We require information from authors about some types of materials, experimental systems and methods used in many studies. Here, indicate whether each material, system or method listed is relevant to your study. If you are not sure if a list item applies to your research, read the appropriate section before selecting a response.

Materials & experimental systems

n/a	Involved in the study	
☒	☐	Antibodies
☒	☐	Eukaryotic cell lines
☒	☐	Palaeontology and archaeology
☒	☐	Animals and other organisms
☐	☒	Clinical data
☒	☐	Dual use research of concern

Methods

n/a	Involved in the study	
☒	☐	ChIP-seq
☒	☐	Flow cytometry
☒	☐	MRI-based neuroimaging

Clinical data

Policy information about clinical studies

All manuscripts should comply with the ICMJE guidelines for publication of clinical research and a completed CONSORT checklist must be included with all submissions.

Clinical trial registration	N/A
Study protocol	N/A
Data collection	For testing polygenic risk scores in the UK Biobank study, all study participant information was collected by UK Biobank in accordance with their ethics approval (North West Multi-Centre Research Ethics Committee). In addition to self-reported disease outcomes and extensive health and life-style questionnaire data, UKB participants are being tracked through their NHS records and national registries (including cause of death and Hospital Episode Statistics).
Outcomes	Primary outcomes for polygenic risk scores were predetermined: Atrial Fibrillation, Stroke, Coronary artery disease, Fascicular block / bundle branch block, atrioventricular block, heart failure, non-ischaemic cardiomyopathy and ventricular arrhythmia. ICD codes for defining these outcomes can be found in supplementary note 2.
